# Letter to the Editor: “Effects of motion sickness patch on postoperative nausea and vomiting in female patients undergoing thyroid surgery: a randomized clinical trial”

**DOI:** 10.1097/JS9.0000000000003456

**Published:** 2025-09-08

**Authors:** Saisai Jing, Yun Li, Jiazhao Song

**Affiliations:** a Department of Oncology, Affiliated Cixi Hospital, Wenzhou Medical University, Cixi, Zhejiang, China; b Experimental Tumorpathology, University Hospital Erlangen, Friedrich-Alexander-Universität Erlangen-Nürnberg, Erlangen, Germany

*Dear Editor*,

We read with interest the study by Yu *et al*^[1]^, a prospective clinical study explored the effectiveness of scopolamine patches in controlling gastrointestinal reactions in post-thyroid surgery patients. It provides great value to the clinical work. In response to this study, we also have some points that we would like to discuss in depth with the authors. This communication has been checked for compliance with the TITAN guidelines^[2]^.

The control group in this study was based on a guideline-standardized protocol rather than a blank/placebo control, making it difficult to isolate the effects of concomitant medications. Other antiemetics and opioid analgesics may have pharmacodynamic interactions with scopolamine (e.g., superimposed sedation, exacerbation of cognitive deficits)^[3]^, thereby underestimating the complexity of real clinical scenarios. It is recommended that future trials use a stratified or crossover design with prespecified stratification and interaction analyses for different concurrent medication scenarios to estimate the independent effects of scopolamine more accurately.

Short-term adverse effects (e.g., a 23.1% incidence of dry mouth) were mainly reported; however, continuation effects after patch removal were not tracked (e.g., blurred vision can last for approximately 24 hours^[4]^). Also, postoperative nausea and vomiting (PONV) was only assessed within 24 hours after surgery, potentially missing delayed nausea and vomiting after 24 hours. Given the central anticholinergic effects of scopolamine, it is prone to drowsiness and disorientation^[4]^. The current study also did not systematically assess postoperative cognitive-related outcomes (e.g., delirium, impaired attention/memory)^[5]^, thus limiting a complete judgment of the benefit-to-risk ratio.

In addition, there is significant individual variation in clinical patients, and a fixed-dose patch (1.5 mg/72 h) is difficult to individualize^[6]^. There is a risk of underexposure or overdose in patients with extremes of body weight, abnormalities of hepatic or renal function, or multiple concurrent doses. In the future, weight/body surface area-based starting doses, risk-stratified dosing timing and patch duration, and the introduction of concentration monitoring or dose-adjustment strategies in high-risk populations may be considered to improve efficacy while reducing the risk of central adverse effects and delirium^[7]^.

## Data Availability

All data generated or analyzed during this study are included in this article, only from “Effects of Motion Sickness Patch on Postoperative Nausea and Vomiting in Female Patients Undergoing Thyroid Surgery: A Randomized Clinical Trial.”
